# Requirements for Human Respiratory Syncytial Virus Glycoproteins in Assembly and Egress from Infected Cells

**DOI:** 10.1155/2011/343408

**Published:** 2011-07-27

**Authors:** Melissa Batonick, Gail W. Wertz

**Affiliations:** Department of Pathology, University of Virginia, MR5 Building, P.O. Box 800904, Charlottesville, VA 22908-0904, USA

## Abstract

Human respiratory syncytial virus (HRSV) is an enveloped RNA virus that assembles and buds from the plasma membrane of infected cells. The ribonucleoprotein complex (RNP) must associate with the viral matrix protein and glycoproteins to form newly infectious particles prior to budding. The viral proteins involved in HRSV assembly and egress are mostly unexplored. We investigated whether the glycoproteins of HRSV were involved in the late stages of viral replication by utilizing recombinant viruses where each individual glycoprotein gene was deleted and replaced with a reporter gene to maintain wild-type levels of gene expression. These engineered viruses allowed us to study the roles of the glycoproteins in assembly and budding in the context of infectious virus. Microscopy data showed that the F glycoprotein was involved in the localization of the glycoproteins with the other viral proteins at the plasma membrane. Biochemical analyses showed that deletion of the F and G proteins affected incorporation of the other viral proteins into budded virions. However, efficient viral release was unaffected by the deletion of any of the glycoproteins individually or in concert. These studies attribute a novel role to the F and G proteins in viral protein localization and assembly.

## 1. Introduction

Human respiratory syncytial virus (HRSV) is the leading viral cause of serious pediatric respiratory tract disease worldwide and a common cause of morbidity in the elderly [1, 2]. Currently there is no vaccine available and the only treatment is a monoclonal antibody given to high-risk infants [3]. Research into vaccine development and therapeutic design is ongoing but an obvious hurdle is the lack of a complete understanding of the replication cycle. The role of the individual viral gene products in each step of virus replication, particularly in the assembly and release of viral particles, is unclear.

HRSV, a member of the *Paramyxoviridae *family, has a negative-sense, single-stranded RNA genome of 15,222 nucleotides. The genome contains ten genes encoding eleven known gene products. The viral ribonucleoprotein (RNP) consists of the RNA genome encapsidated by the nucleoprotein (N), having associated the phosphoprotein (P) and RNA-dependent RNA polymerase (L) [4, 5] as well as the M2-1 protein, which is involved in transcription processivity [6]. The viral genome also encodes the structural matrix protein (M) [7] and three transmembrane glycoproteins that are presented on the surface of the viral particle, the small hydrophobic glycoprotein (SH) [8], the attachment glycoprotein (G) [9], and the fusion glycoprotein (F) [10]. The major identified function for the glycoproteins is in viral entry. The F protein is required for fusion between the cellular and viral membranes [11], thus allowing the viral genome to enter the host cell cytoplasm. The G protein is involved in host cell attachment [12] and is necessary for infectivity *in vivo *and in some cultured cell types while in others its deletion has no effect on infectivity [13]. The role of the SH protein is still unclear, although it has recently been found to inhibit apoptosis [14, 15]. The viral glycoproteins along with the M, N, P, L, and M2 proteins are essential structural and enzymatic components of HRSV. How these viral components assemble to form a newly infectious virion and how the release of the virus from the host cell is coordinated is largely unknown.

HRSV assembles at and buds from the plasma membrane of the infected cell to gain its envelope [16–18]. For many paramyxoviruses, it has been shown that the M protein is sufficient for particle release, and in some cases the F glycoprotein has been shown to enhance the process [19–24]. In other instances, such as for SV5, viral budding requires the M protein, one of its two viral glycoproteins, and the N protein [25]. These studies used transient transfection systems to additively express individual viral proteins and analyzed their effects on the amount of released virus-like particles (VLPs). A similar study done with HRSV found the M, P, N, and F proteins to be the minimal requirements for formation and passage of VLPs containing minigenomes [26]. However, this study did not look specifically at the release of the VLPs; therefore it is still unclear whether the F protein is needed only for viral entry or for entry and subsequent steps leading to VLP passage.

Previously, we examined the effect of HRSV glycoprotein deletions on the directional targeting of the virus in polarized epithelial cells [27]. This earlier study was a qualitative investigation which demonstrated that HRSV particles are able to bud directionally in the absence of all three glycoproteins. However, a quantitative analysis of the contribution of these viral proteins to the late stages of viral protein trafficking, assembly, and release was not done. In the present study, the role of the three viral glycoproteins in viral assembly and egress from infected cells was examined. First, the contribution of each glycoprotein to viral assembly was analyzed by investigating the localization of the remaining viral structural proteins at the plasma membrane by confocal microscopy. Next, the protein composition of particles released from cells infected with WT or glycoprotein deleted HRSV was biochemically quantitated to determine if one or more glycoproteins are involved in the incorporation of viral proteins into newly formed viral particles. Finally, the effect of each glycoprotein on the efficiency of virus particle budding from infected cells was analyzed.

## 2. Results

### 2.1. Engineered Viruses

**Figure 1:**
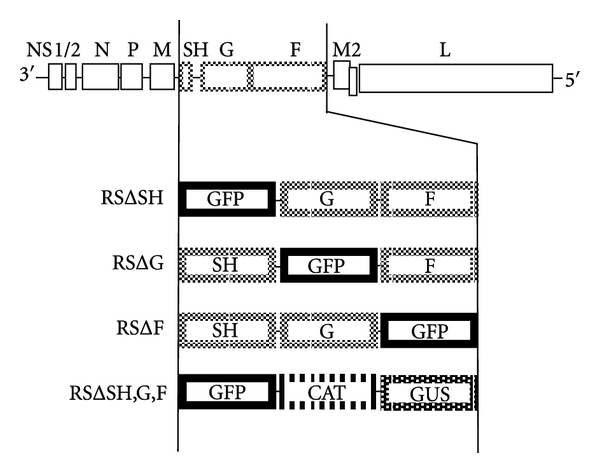
Schematic of the gene content of engineered viruses. All engineered viruses were generated from a cDNA of the A2 strain of HRSV. Viruses RSΔSH, RSΔG, and RSΔF are missing the ORFs of the SH, G, and F genes, respectively, which have been substituted with that of GFP. Virus RSΔSH,G,F has the ORFs encoding each of the three HSRV glycoproteins replaced with those of reporter genes GFP, CAT, and GUS, respectively.

To examine the involvement of each glycoprotein in the assembly and release of HRSV, we used viruses previously engineered to have each glycoprotein gene deleted individually from the HRSV genome and its ORF replaced with that of a reporter gene (Figure 1) [27]. We also utilized an engineered virus with all three glycoprotein genes deleted [27]. Since HRSV transcription is obligatorily sequential and due to attenuation at each gene junction, the replacement of any deleted genes was necessary to maintain authentic levels of viral gene expression. Briefly, in the case of individual deletions, GFP was used to replace the deleted gene. When three genes, SH, G, and F, were deleted they were replaced respectively with the GFP, CAT, and GUS, reporter genes (Figure 1). All of the viruses described above not only maintained the same number of genes as in WT HRSV but also had the genuine intergenic junctions to preserve authentic transcription levels.

### 2.2. Effect of Single Glycoprotein Deletions on the Intracellular Localization of HRSV Proteins at the Site of Viral Assembly

The HRSV proteins must assemble at the plasma membrane to initiate budding of the RNP to form newly enveloped and infectious viral particles upon release from the cell. In polarized epithelial cells, this process takes place on the apical membrane [18]. We previously demonstrated that the glycoproteins are not involved in the directional targeting of the viral proteins for viral release from the apical membrane to occur [27]. However, the techniques used in this prior study did not analyze the effects of individual glycoprotein gene deletions on intracellular assembly with other viral proteins. To address this question, the level of colocalization at the plasma membrane of each of the three glycoproteins with the N protein, which was used as a marker for ribonucleocapsids, was analyzed by microscopy. 

**Figure 2:**
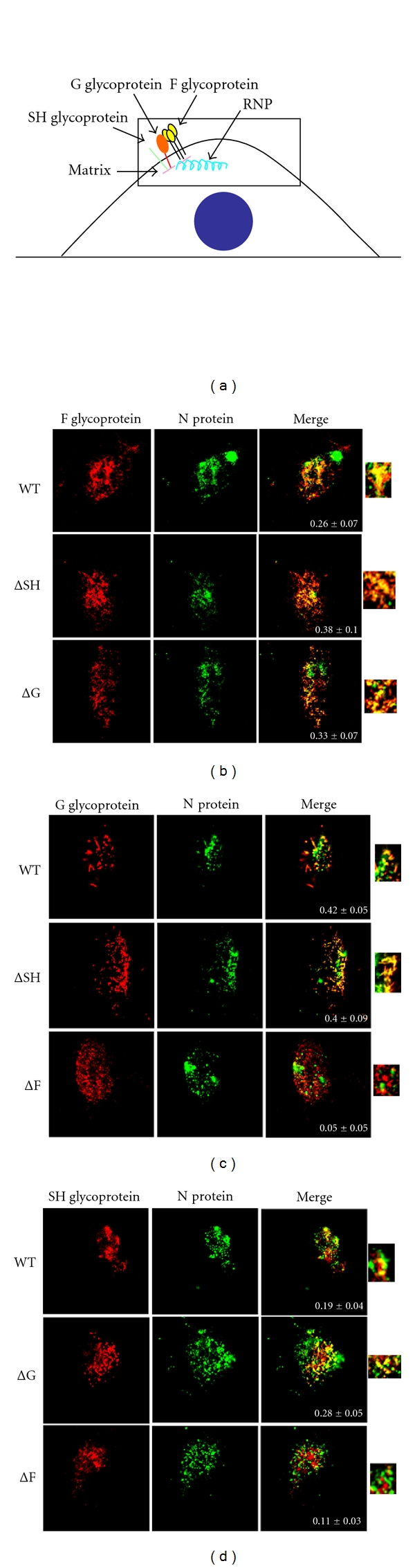
Effect of the deletion of individual glycoprotein genes on colocalization of the HRSV nucleocapsid protein with each glycoprotein at the plasma membrane. (a) A diagram of an infected cell depicting HRSV assembly at the plasma membrane. The boxed area represents the 1 *μ*m *z*-stack shown in subsequent confocal microscope images. The nucleus is depicted as a blue circle. A549 cells infected with WT or glycoprotein deleted HRSV at a MOI of 0.2 were fixed, permeabilized, and incubated with an anti-N antibody and an anti-F antibody (b), an anti-G antibody (c), or an anti-SH antibody (d). The glycoprotein stains are shown in the left row (red), the N protein stains in the middle row (green), and a merge of the two stains in the right row. A blowup of the merge is also shown. Images were taken on a Zeiss confocal microscope. An average of fourteen cells were imaged per staining, and colocalization was measured using ImageJ JACoP plugin. Pearson's coefficient ± standard error of mean is shown within the merge panels.

A549 cells were infected with WT or individual glycoprotein deleted viruses at a MOI of 0.2. Twenty-four hours after infection the cells were fixed, permeabilized, and incubated with primary antibodies against N, and SH, or G, or F proteins in turn and followed by secondary antibodies conjugated to AlexaFluor 647 and 594 fluorescent dyes. A confocal microscope was used to take multiple *z* plane images (*z*-stacks) through the cell as described in Section 4. Since viral assembly takes place at the plasma membrane we focused on the *z*-stack containing that section of the infected cell, which is shown in all images and is depicted in the diagram in Figure 2(a). In Figures 2(b), 2(c), and 2(d) the N protein is shown in green with the three glycoproteins, in individual panels, shown in red (F and N staining is shown in Figure 2(b), G and N staining is shown in Figure 2(c), and SH and N staining is shown in Figure 2(d)). A merge panel of the two stained proteins is also shown along with a magnification of the merge to further demonstrate the colocalization in each image. Also shown within each merge panel is the amount of colocalization quantitated by NIH ImageJ software [28] and is depicted as Pearson's coefficient [29], where a number near +1 suggests perfect correlation between two biomolecules, a number near 0 indicates no correlation, and a number near −1 suggests an inverse correlation or exclusion of the biomolecules.

An average of fourteen cells were imaged per staining, and their average Pearson's coefficient was quantitated along with the standard error of the mean (SEM). As shown in Figure 2(b), similar levels of colocalization were observed between the N and F proteins in cells infected with WT, ΔSH, and ΔG viruses (Pearson's coefficients of 0.26, 0.38, and 0.33, resp.) suggesting that the SH and G proteins are not necessary for the assembly of the remaining glycoproteins and the RNPs at the cell surface. In Figure 2(c) similar amounts of colocalization between the N and G proteins were observed in cells infected with WT and ΔSH viruses (Pearson's coefficient of 0.42 and 0.40, resp.) whereas a ninefold decrease in colocalization between the N and G proteins was observed in cells infected with ΔF virus (Pearson's coefficient of 0.05). This indicated that F is involved in the colocalization of the N and G proteins at the cell surface. In addition, the distribution of the G protein at the plasma membrane differed in cells infected with ΔF virus as compared to cells infected with WT or ΔSH viruses. In the absence of the F glycoprotein, G protein had a more monodisperse distribution rather than the filamentous forms seen in WT-infected cells (cf. Figure 2(c), cells infected with WT virus and ΔF virus). Colocalization was observed between the N and SH proteins in cells infected with WT virus, albeit at a relatively low level (Figure 2(d); Pearson's coefficient of 0.19). A slightly increased but similarly low level of colocalization between the N and SH proteins was seen in cells infected with ΔG virus (Pearson's coefficient of 0.28). In cells infected with the ΔF virus, however, colocalization was seen at approximately half of WT levels (Pearson's coefficient of 0.11). Thus, the above results show that the absence of the F protein affects the colocalization of the G protein with the N protein at the plasma membrane and, to a lesser extent, that the absence of the F protein affects the colocalization of the SH protein with the N protein at the plasma membrane.

**Figure 3:**
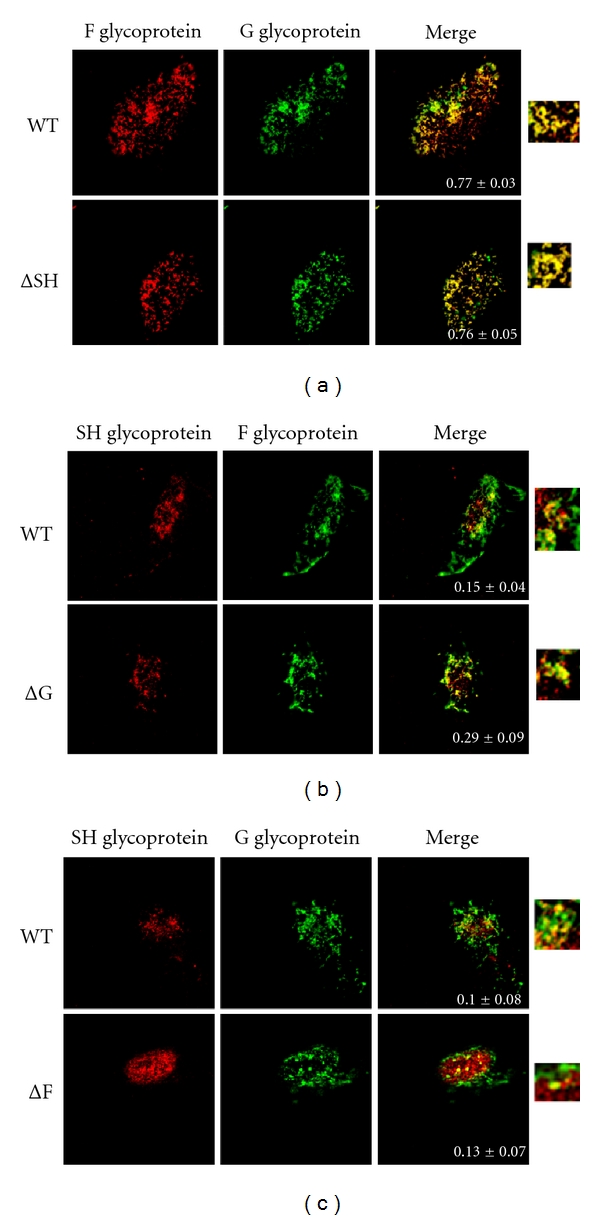
Effect of the deletion of individual glycoprotein genes on the colocalization of pairs of HRSV glycoproteins at the plasma membrane. Localization of HRSV glycoproteins at the site of viral assembly by confocal microscopy. A549 cells infected with WT or glycoprotein deleted HRSV as indicated at a MOI 0.2 were fixed, permeabilized, and incubated with primary antibodies against two different glycoproteins. (a) Staining of F and G glycoproteins where F is shown in red and G in green. (b) Staining of SH and F where SH is shown in red and F in green. (c) Staining of SH and G where SH is shown in red and G in green. A merge of the two stains is shown in the right row. A blowup of the merge is also shown. Images were taken on a Zeiss confocal microscope. An average of twelve cells were imaged per staining, and colocalization was measured using ImageJ and JACoP plugin. Pearson's coefficient ± standard error of mean is shown within the merge panels.

Next, we examined the effect of deletions of individual glycoproteins on the levels of colocalization between the remaining two glycoproteins at the plasma membrane by microscopy. A549 cells infected with WT or engineered viruses were fixed and permeabilized and then incubated with primary antibodies against two of the glycoproteins. In Figure 3(a), the F glycoprotein is shown in red and the G protein is shown in green. In Figures 3(b) and 3(c), the SH glycoprotein is shown in red while the F and G proteins are shown in green, respectively. In each case a merge panel of the two proteins is also shown along with a magnification of the merge to further illustrate the extent of colocalization in each image. Also shown within the merge panel is the amount of colocalization depicted as Pearson's coefficient as described for Figure 2. An average of twelve cells were imaged per staining, and their colocalization is shown along with the standard error of the mean (SEM).

High levels of colocalization were observed between the F and G glycoproteins in cells infected with WT virus (Figure 3(a); Pearson's coefficient of 0.77), and deletion of the SH gene did not affect F and G protein colocalization (Figure 3(a); Pearson's coefficient of 0.76). Colocalization between the SH and F proteins in WT- or ΔG-infected cells was observed at relatively low levels (Pearson's coefficient of 0.15 and 0.29, resp.; Figure 3(b)). Low amounts of colocalization were also observed between the SH and G proteins in WT- and in ΔF-infected cells (Pearson's coefficient of 0.10 and 0.13, resp.; Figure 3(c)). Taken together these data indicate that deletion of any single glycoprotein did not affect the localization of the other two glycoproteins. Further, these data show the F and G glycoproteins colocalize substantially; however, SH does not colocalize with either F or G proteins to a high degree.

### 2.3. Effect of HRSV Glycoprotein Deletions on the Incorporation of Viral Proteins into Newly Budded Virions

**Figure 4:**
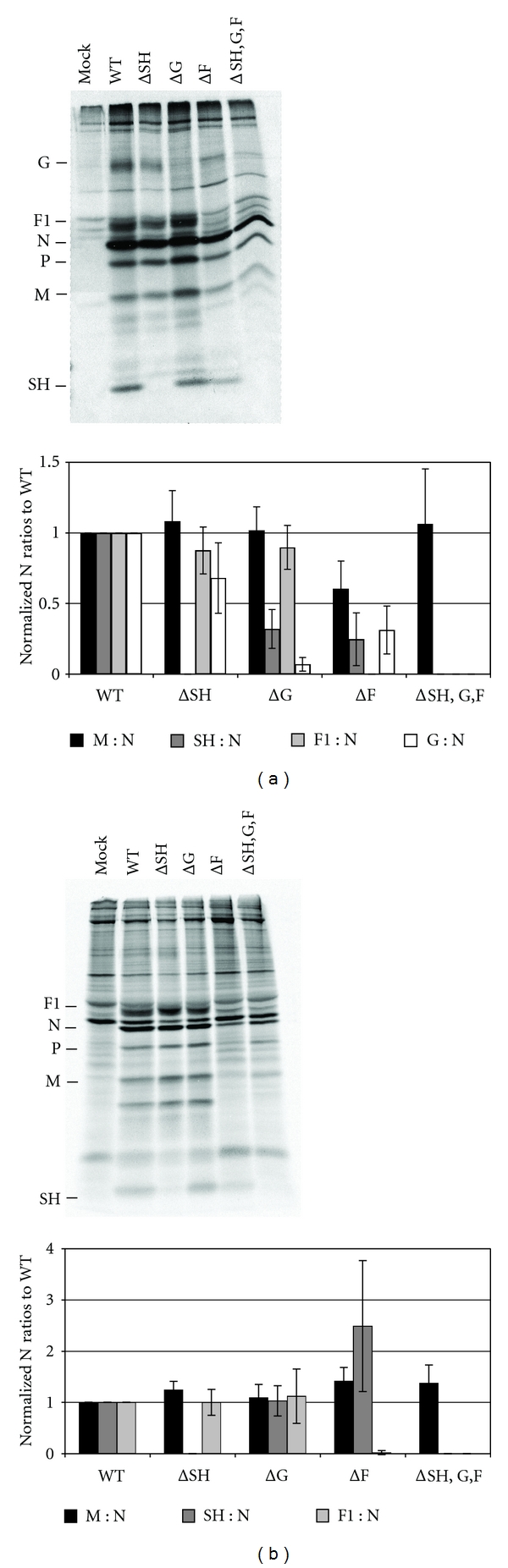
Effect of deletion of glycoprotein genes on the incorporation of HRSV proteins into released viral particles. Twenty-four hours after infection, A549 cells infected with WT or glycoprotein deleted HRSV at a MOI of 1.0 were radiolabeled with ^35^S Cys/Met for 16 h. The supernatant containing the released viral particles was collected, cleared of cell debris, and concentrated by centrifugation. (a) The viral pellet was resuspended, and viral proteins were immunoprecipitated with a polyclonal anti-HRSV antibody along with monoclonal antibodies against G and SH. Proteins were analyzed by electrophoresis on a 12% SDS polyacrylamide gel. A representative gel is shown. The bar graph depicts ratios of each HRSV structural protein to N protein, normalized to the ratios found in WT viral particles. (b) Infected cells were lysed and viral proteins were immunoprecipitated with a polyclonal anti-HRSV antibody along with antibodies against G and SH. Proteins were analyzed on a 12% SDS-PAGE. A representative gel is shown. The bar graph depicts ratios of each HRSV structural protein to N protein, normalized to the ratios found in WT-infected lysate. Proteins were quantitated on a phosphorImager. Error bars represent standard deviation from at least three experiments.

To further scrutinize a potential role for HRSV glycoproteins in the late stages of the viral replication cycle, released particles were biochemically analyzed for their viral protein composition. A549 cells were infected with WT or glycoprotein deleted HRSV at a MOI of 1.0. Twenty-four hours after infection the cells were radiolabeled with ^35^S Cys/Met for 16 h. The supernatant containing the released virions was collected and concentrated by centrifugation. The viral proteins present in the pelleted virions were analyzed by immunoprecipitation followed by SDS polyacrylamide gel electrophoresis as described in Section 4. The ratio of the HRSV structural proteins, M, F, G, and SH relative to the N protein was determined. The ratios found in glycoprotein deleted viral particles were compared to those found in WT viral particles (Figure 4(a)). The F glycoprotein is translated as the precursor protein F0 and then cleaved into F1 and F2. The cleaved product F1 was labeled most efficiently by ^35^S Cys/Met, and so we quantified this protein to represent F. As shown in Figure 4(a) when cells were infected with the **Δ**SH virus no substantial differences in the incorporation of the M, G, or F proteins into viral particles were observed when compared to WT particles. Cells infected with the **Δ**G virus had WT-like levels of the M and F proteins in released virions; however, the amount of SH protein incorporation was decreased to approximately 40% of that found in WT viral particles. Virus released from **Δ**F-infected A549 cells had slightly decreased levels of the M protein, at 70% of that found in WT virus. The most dramatic differences in particles released from **Δ**F-infected cells were observed in the amounts of incorporated SH and G proteins, which were reduced to 25% and 30% of WT levels, respectively. Cells infected with the **Δ**SH,G,F virus had WT-like amounts of M protein incorporation.

Although all engineered viruses containing glycoprotein deletions had replacement reporter genes to ensure WT levels of transcription of the downstream genes [30], viral protein levels in cells infected with the engineered viruses were also analyzed to determine whether the levels of protein synthesis were affected by the deletion of the glycoproteins. To confirm that the protein incorporation defects observed in budded viral particles were not due to decreased amounts of those viral proteins in infected cells, the HRSV structural proteins in infected cell lysates were also quantitated and reported as a ratio to the N protein. As shown in Figure 4(b), the ratios of the M, SH, and F proteins to the N protein in cell lysates infected with glycoprotein deleted viruses were equivalent to or greater than those found in WT-virus-infected cell lysate. These data indicated that the effect of G and F glycoprotein deletions on decreased SH incorporation into particles was not due to overall reduction in SH protein synthesis.

We used A549 cells in these studies because infectivity experiments performed in different cell types showed that the ΔF virus, which utilizes GP64 for viral entry [31] did not give a productive infection in HEp2 cells (see supplementary Figure 1(a) in Supplementary Material available online at doi: 10.1155/2011/343408). We found that A549 cells allowed for more robust infection of ΔF viruses than HEp2 and Vbac cells [32] (supplementary Figure 1(a)). In contrast, the ΔSH virus had equivalent levels of infection in all cell types tested (supplementary Figure 1(b)). However, in A549 cells the quantitative detection of a discrete band of a mature 84 kD G protein even in WT-infected cell lysates was difficult. We suspect, in A549 cells, that the heterodisperse nature of the extensive O-linked glycosylation and the low methionine content of G protein prevent detection of a discrete band. However, previous work showed that gene transcription levels were not altered in the glycoprotein deleted viruses [30], and we know from the microscopy data in this study that the amount of the G protein stainings in WT- and in ΔF-infected A549 cells were comparable (Figures 2(c) and 3(c)). As such, although we were unable to confirm equivalent G-to-N-protein ratios in cell lysates infected with glycoprotein deleted viruses as compared to WT-infected cell lysates (Figure 4(b)), data strongly suggests that the G protein synthesis was unaffected by the deletion of the F glycoprotein. 

### 2.4. Involvement of HRSV Glycoproteins in Viral Release

**Figure 5:**
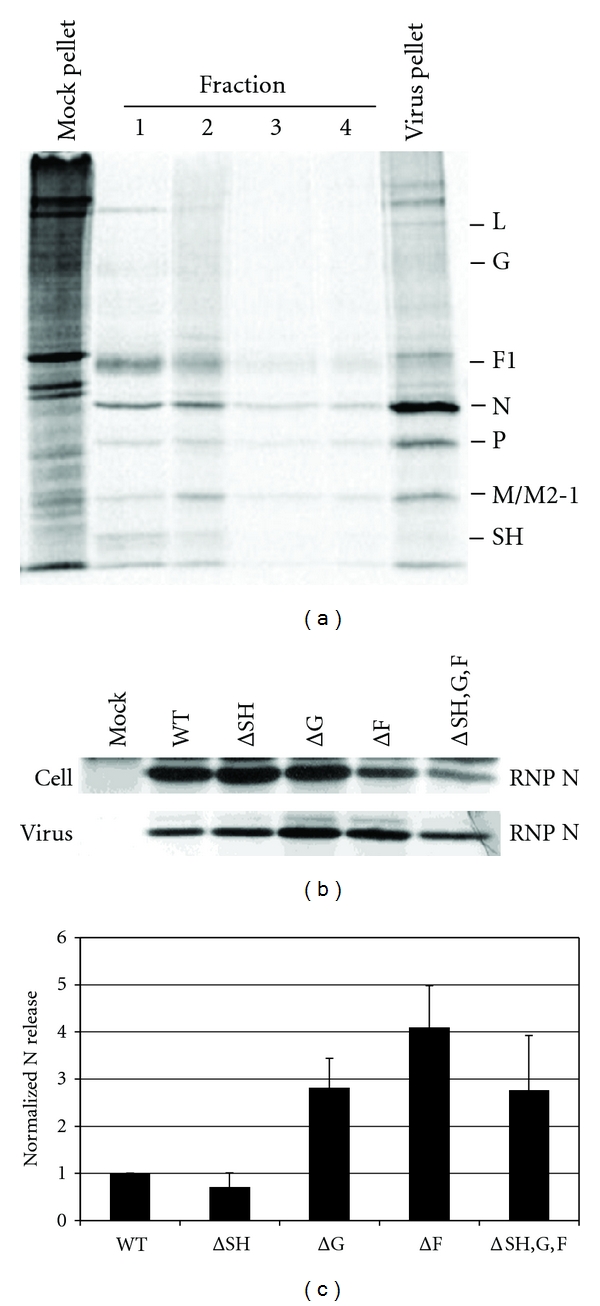
Effect of glycoprotein deletions on the release of virions from infected A549 cells. A549 cells were infected with WT or glycoprotein deleted virus and particles released into the supernatant were quantified as a percent of total virus produced. The N protein in isolated RNPs was used to quantitate the virus in cell lysates and in virions. (a) Separation of RNPs containing the viral genome and associated proteins from soluble proteins in infected cell lysates. Infected cell lysates were ultracentrifuged through a 40% glycerol cushion. Fractions were taken from the top of the tube (fraction 1) to the bottom of the tube (fraction 4) in 0.5 mL volumes. The pellet was resuspended in 0.1 mL lysis buffer. Viral proteins in each fraction and the pellet were immunoprecipitated and analyzed on a 12% SDS polyacrylamide gel. A representative gel is shown. HRSV proteins are indicated on the right of the gel. (b) Ribonucleoprotein (RNP) associated N protein in infected cell lysates was harvested as described above and in Section 4, and viral proteins were analyzed by immunoprecipitation and electrophoresis on 12% SDS gels. Released virions were recovered from supernatant fluids by centrifugation and viral proteins were analyzed by immunoprecipitation and electrophoresis on 12% SDS gels. A representative gel is shown. (c) Bar graph depicts the percent of N protein quantified in released virus from total RNP-associated N protein quantified in infected cell lysate and in released virus. The N protein was quantitated by phosphoimage analysis. Error bars represent standard deviation from at least three experiments.

Since we observed a role for the glycoproteins in viral assembly, we asked if they were also involved in the other late stage of the replication cycle, viral release. We knew from our previous work that the glycoproteins were not required for viral release [27], but those studies had not quantitatively examined the effect of glycoprotein deletions on the efficiency of viral budding. To address this question, we infected A549 cells with either WT or glycoprotein deleted virus and quantified the amount of particles released as a percent of total virus produced in cells. In infected cells the N protein will either associate with the viral genome to form RNPs and localize to the plasma membrane for viral assembly or remain in the cytoplasm as a soluble protein. To quantify HRSV production and viral release, we only considered the N protein bound to RNPs since this is the form that assembles into viral particles and buds from the plasma membrane. Therefore our first step was to separate N protein in RNPs from free soluble N protein in infected cell lysates as a measure of virus inside the cell. Infected A549 cells were radiolabeled with ^35^S Cys/Met and lysed as described in Section 4. The lysate was layered on top of a 40% glycerol cushion and ultracentrifuged as described in Section 4. Fractions were taken from the top of the tube (fraction 1) to the bottom of the tube (fraction 4), and the pellet was resuspended in lysis buffer. HRSV proteins in each fraction were identified by immunoprecipitation followed by analysis on 12% SDS polyacrylamide gel as described in Section 4. As shown in Figure 5(a), soluble proteins such as G, F1, N, P, M, and SH were found in fractions 1–4 whereas predominantly proteins bound to the viral genome, L, N, P, and M2-1, were found in the pellet.

To quantify the amount of virus released from infected cells, A549 cells were infected with WT or glycoprotein deleted viruses at a MOI of 1.0. Twenty-four hours after infection the cells were radiolabeled with ^35^S Cys/Met. Supernatants containing released virions were collected and concentrated by centrifugation, as described previously in [27] and in Section 4. Pelleted virions were disrupted and HRSV proteins were identified by immunoprecipitation followed by analysis by polyacrylamide gel electrophoresis. The infected cells were also lysed and RNPs separated by glycerol sedimentation as described above. The RNP bound N protein present in pelleted virions and in infected cell lysates was analyzed on SDS gels and quantitated. The amount of N protein present in released virus is shown as a percent of total N protein in RNPs quantified from both lysate and virus (Figures 5(b) and 5(c)). Cells infected with ΔSH, ΔG, ΔF viruses, or the triple deletion virus ΔSH,G,F, did not show any decrease in the levels of released viral particles compared with cells infected with WT virus. These findings show that the viral glycoproteins do not negatively affect the efficiency of viral egress.

## 3. Discussion

The major HRSV structural proteins are the nucleocapsid protein (N), the matrix (M) protein, and the F, G, and SH glycoproteins, all of which associate at the plasma membrane for viral assembly and release. Little is known about the protein requirements of HRSV during the late stage of virus replication. It has been established for many paramyxoviruses that the M protein is necessary for the formation of viral particles [19–25], but the involvement of the glycoproteins varies depending upon the virus. The HRSV glycoproteins have defined roles in attachment and entry, but delineating their potential roles in the downstream steps of the replication cycle is difficult due to the necessity of the F protein for viral entry into cells. 

In this study we utilized genetically engineered HRSV in which one or more of the glycoprotein genes were deleted and replaced with reporter genes (Figure 1). Recombinant viruses lacking the F protein were used in conjunction with a cell line that expresses GP64 to overcome the inherent viral entry deficit of a F deleted virus [32]. In a previous study using these engineered viruses we showed that HRSV with all three glycoprotein genes deleted released viral particles specifically from the apical membrane of polarized epithelial cells [27], which confirmed that the glycoproteins were not essential for directional targeting or for viral release. However, we did not address whether the glycoproteins affected the quantitative efficiency of viral egress or whether the glycoproteins were involved in the assembly of the other viral proteins at the plasma membrane to form newly infectious viral particles.

Prior to viral budding from infected cells, the three HRSV glycoproteins must assemble with the RNPs at the plasma membrane. To determine if the individual glycoproteins are involved in the assembly of the other structural proteins, we studied cells infected with viruses having individual glycoprotein genes deleted and examined the colocalization of the remaining glycoproteins at the plasma membrane by confocal microscopy. We observed that the deletion of any individual glycoprotein did not affect the colocalization of the remaining glycoproteins at the cell surface (Figure 3). Using the N protein as a marker for RNPs, we also investigated whether deletion of the individual glycoproteins affected the assembly of the remaining glycoproteins and the N protein at the plasma membrane. We found that deletion of the F glycoprotein substantially reduced the colocalization of the G protein with the N protein at the plasma membrane. Deletion of the F protein also reduced colocalization between the SH and N proteins at the cell surface (Figures 2(c) and 2(d), resp.). These data indicate that F protein plays a previously unidentified role in localizing virus structural proteins during HRSV assembly. Interestingly, we also observed in ΔF-virus-infected cells that the staining pattern of G protein on the plasma membrane was altered as compared to WT-virus-infected cells (Figure 2(c), compare G staining in WT-infected cell with that in ΔF-infected cell). These findings are consistent with results from a previous study which showed that the G glycoprotein localized to viral filaments at the plasma membrane in cells infected with WT virus, but not in cells infected with an engineered HRSV with the cytoplasmic tail of F protein deleted (FΔCT) [33], further suggesting that the F protein is involved in protein sorting at the cell surface.

Once the viral proteins have assembled at the cell surface, they are incorporated into newly formed virus particles as RNPs bud from the plasma membrane. We analyzed whether deletion of each individual glycoprotein affected the ability of the remaining structural proteins to incorporate into budded virions. We found that cells infected with viruses lacking the F glycoprotein incorporated decreased amounts of the G and SH proteins relative to the N protein in released virus (Figure 4(a)). These findings are in agreement with the previous observation that the deletion of the F protein resulted in decreased amounts of colocalization between RNPs and both the G and SH proteins at the cell surface (Figures 2(c) and 2(d), resp.). Cells infected with viruses lacking the G protein also incorporated lower amounts of the SH protein into released virions (Figure 4(a)). These results indicate that the F and G glycoproteins are involved in HRSV particle assembly. 

We used two approaches, microscopy and protein biochemistry, to investigate the effect of the glycoprotein deletions on viral assembly. These two methods further dissected the assembly process into two separate steps: the accumulation of viral proteins at the plasma membrane, which is the site of viral assembly, and the incorporation of the accumulating proteins into budding viral particles. Both experiments concluded that SH is not involved in either stage of viral assembly as WT-like levels of colocalization were observed between the remaining structural proteins at the plasma membrane of ΔSH-infected cells, and WT-like levels of HRSV proteins were found in released particles. Both the microscopy and the biochemical data indicated that F glycoprotein is involved in both steps of the assembly process as colocalization between the N and SH proteins and between the N and G proteins in ΔF-infected cells was decreased as compared to WT-infected cells. Cells infected with ΔF virus also had decreased amounts of the G and SH proteins incorporated into budded virions as compared to WT particles. The G glycoprotein does not appear to be implicated in the accumulation of viral proteins at the plasma membrane since WT and elevated levels of colocalization were observed between the N, SH, and F proteins in cells infected with ΔG virus. However, the G protein was involved in the incorporation of the SH protein into the budding virion as shown by a decrease in the SH-to-N-protein ratio in particles released from ΔG-infected cells as compared to WT virions. The reasons for this dichotomy are unknown. The SH protein exists in several modified and unmodified forms including its modification by polylactosaminoglycans, and these forms as well as the unmodified form are found in virions [34]. It would be of interest to determine whether there may be interactions between one or more of the modified forms of SH and the highly O glycosylated G protein. The M protein was incorporated at near WT-like levels in all glycoprotein deleted viruses, including the triple deleted virus, indicating that the M protein assembles into budding virus independently of the glycoproteins.

The final step to produce new viral particles after the viral proteins have assembled at the plasma membrane is egress from infected cells. To determine if the viral glycoproteins were involved in the budding process, we analyzed whether deletion of the glycoproteins affected the efficiency of viral release. We found that the absence of any or all of the three glycoproteins did not have a deleterious effect on the amount of virus released from infected cells (Figure 5). Indeed, ΔF- and ΔG-infected cells released slightly increased amounts of viral particles into the supernatant as compared to WT infected cells. We speculate that fewer glycosylated proteins in the virus led to less reattachment of viral particles to the cell. This resulted in fewer cell-associated virions and hence an increase in released viral particles in the supernatant. 

Previous studies have shown an interaction between the SH and G proteins in cells infected with WT HRSV [35] which may explain the requirement for the G protein in the incorporation of the SH protein into budding virus. Interactions have also been shown for the G and M proteins [36] and for the G and F proteins [37]. An oligomeric complex was also reported in infected cell lysates between the F, G, and SH proteins [38]. It has been hypothesized that the F glycoprotein interacts with other viral proteins via its cytoplasmic tail (CT), based on a study which showed that the ability of the F protein to interact with lipid rafts at the plasma membrane was disrupted and the localization of the F and the G proteins at the plasma membrane was altered when cells were infected with FΔCT virus [33]. These previous studies along with the data presented here indicate that the three HRSV glycoproteins interact with other viral structural proteins in infected cells, and, importantly, this study attributes a novel role to the F and G glycoproteins in the late stages of viral replication.

## 4. Materials and Methods

### 4.1. Cells and Antibodies

A549, Vero, and HEp2 cells were acquired from the American Type Culture Collection (ATCC). A549 cells were grown in Ham's F12K medium (Sigma Aldrich, St. Louis, Mo) containing 10% fetal bovine serum, and Vero and HEp2 cells were grown in Dulbecco's minimal essential medium (DMEM; Invitrogen, Carlsbad, Calif) containing 5% fetal bovine serum. Vbac cells (Vero cells expressing the baculovirus GP64 protein carrying the HRSV F protein COOH-terminal residues 563 to 573) were described previously in [32]. Monoclonal antibodies (MAbs) 19 and 29 were provided by Geraldine Taylor (Institute for Animal Health, Compton, UK), as was the bovine polyclonal antibody against all HRSV proteins, R45. The monoclonal antibody, mAb15, was provided by James Stott (Institute for Animal Health, Compton, UK). Rabbit anti-SH antibody was provided by Biao He (University of Georgia). AlexaFluor-conjugated secondary antibodies were from Molecular Probes (Carlsbad, Calif).

### 4.2. Construction of cDNAs and Recovery of Infectious HRSVs

 All cDNAs were constructed from the HRSV A2 strain. cDNAs engineered to contain glycoprotein gene deletions for these studies were constructed as described previously in [27, 30, 33]. Briefly, using standard cloning techniques, vectors were constructed lacking the G or F transmembrane glycoprotein open reading frame (ORF) and containing the EGFP ORF instead, as shown in Figure 1. All HRSV ORFs were maintained in their original genome positions to maintain expression profiles similar to that of a wild-type virus. For generation of the recombinant wild-type (WT) virus, the vector contained the authentic SH, G, and F ORFs and no marker protein ORF. The ORFs contained in each of the vectors were separated by authentic HRSV intergenic junctions and flanked by unique restriction sites *Fse*I and *Asc*I. Using matching *Fse*I and *Asc*I restriction sites, the vectors were then cloned into an SH/G/F-deleted cDNA backbone. The engineered cDNAs were screened by restriction enzyme analysis, and all modified areas were verified by nucleotide sequencing prior to virus recovery.

Infectious viruses were recovered from cDNA as described previously in [31]. Notably, to relieve selection pressure that might result from the effect of gene replacements, a plasmid encoding a chimeric VSV G protein was included in the initial transfection, and Vbac cells were used for virus amplification. Viral RNAs were harvested from cells infected with the engineered viruses at passage 3, amplified by RT-PCR, and the sequence of selected areas was verified by bulk nucleotide sequence analysis. No changes were found. Virus stocks at passages 3 to 5 were used for the experiments described.

### 4.3. Virus Infections

Infections of cells by HRSV were carried out by adsorbing virus to cells for 1.5 h at 33°C, followed by removing the inoculum and washing the cells once with growth media and then continuing incubation at 33°C for 24 h or 48 h, depending on the assay.

### 4.4. Immunofluorescence Staining and Confocal Microscopy

A549 cells were plated on sterile coverslips in 6-well plates (BD biosciences, San Jose, Calif). Cells were then infected with engineered viruses at a MOI of 0.2 and further incubated at 33°C. Twenty-four hours after infection, cells were washed twice with PBS and fixed with 3.7% paraformaldehyde (PFA) in phosphate-buffered saline (PBS) for 15 min at room temperature (RT). Cells were washed twice with PBS, permeabilized with 0.02% triton X-100, blocked in 1% BSA for 10 min, and stained with the following primary antibodies diluted in 0.1% BSA: F mAb19 at 1 : 5000; G mAb29 at 1 : 2000; N mAb15 at 1 : 15000; rabbit anti-SH at 1 : 200 for 1 h at RT. Cells were washed twice with PBS, blocked with 1% BSA, and incubated with secondary antimouse or antirabbit antibodies conjugated to AlexaFluor 594 or 647 (Invitrogen) at a dilution of 1 : 1000 for 30 min at RT. When costaining with primary antibodies from the same host, the Apex labeling kit was used (Invitrogen) to directly conjugate the primary antibody with AlexaFluor 647. Cells were washed twice with PBS and stained with Hoechst stain (Molecular Probes) at 0.05 mg/mL in PBS. Cells were washed three times with PBS, mounted on slides, and stored at 4°C in the dark. Images were taken on a Zeiss LSM 510-UV confocal microscope using the 40x or the 100x objective. 1 *μ*m *z*-stacks were taken through each cell, and the top plasma membrane stack was taken to visualize the site of HRSV assembly. The plasma membrane containing section was identified as the most apical stack containing in-focus staining and without visible nuclear staining.

The fluorescence in the top plasma membrane stack was quantitated to determine the levels of colocalization between N and each glycoprotein or between two glycoproteins. Quantitation of pixels was done using ImageJ with JACoP plugin [39]. Thresholds for each wavelength were determined on JACop using Costes automatic thresholding [40], and the amount of colocalization of pixels above the threshold was determined and reported as Pearson's coefficient (*R*) ± standard error of the mean (SEM).

### 4.5. Viral Assembly and Release Assays

A549 cells were plated in 60 mm dishes (BD biosciences) and incubated at 37°C. Approximately 6 h later, the cells were infected with engineered viruses at a MOI of 1.0 and incubated at 33°C. 24 h later the cells were radiolabeled using ^35^S Cys/Met trans mix (Promega, Madison, Wis) and incubated at 33°C. 16 h later the supernatants containing the released virions were carefully removed from the cell monolayer and put into 2 mL tubes. The supernatants were centrifuged at 750 ×g for 4 min to pellet any cell debris. The supernatants containing the viral particles were transferred to a new 1.5 mL tube and centrifuged at 1500 ×g for 30 min, a speed that allows for the fragile HRSV particles to be concentrated and still maintain infectivity [27]. We confirmed that this pelleting method concentrated virions with one glycoprotein or two glycoproteins deleted to ensure that particles with decreased density still pelleted at the low speed (data not shown). The viral pellets were resuspended in 0.1 mL lysis buffer consisting of 1% NP40, 0.4% deoxycholic acid, 66 mM EDTA, and 10 mM Tris-HCl, pH 7.4. The entire volume was incubated with the bovine polyclonal antibody R45 at 1 : 100 dilution + rabbit anti-SH antibody at 1 : 25 + anti-G mAb29 at 1 : 100 overnight at 4°C. Meanwhile, the infected cell monolayers were washed once with PBS and then lysed in lysis buffer (described above). The lysates were harvested into 1.5 mL tubes, incubated on ice for 7 min, and vortexed for 30 sec. The cell nuclei were removed by centrifugation at 14,000 rpm for 1 min. The RNP-associated proteins were then pelleted by ultracentrifugation through a 40% glycerol cushion at 190,000 ×g for 2 h at 4°C. The pellets were resuspended in 0.1 mL lysis buffer and incubated with R45 antibody at 1 : 100 dilution overnight at 4°C. The next day 25 *μ*L of Protein G sepharose (GE Healthcare, Piscataway, NJ) was added to each lysate + antibody and released virus + antibody and incubated for at least 1 h at 4°C. The immunoprecipitations were washed 3x with wash buffer consisting of 1% NP-40, 0.5% DOC, 0.1% SDS, 150 mM NaCl, and 10 mM Tris-HCl, pH 7.4, and then boiled for 3 min in *25* *μ*L of 2x reducing Laemmli buffer. All samples were electrophoresed on 12% polyacrylamide gels. The gels were fixed, dried, and exposed to both film and a phosphorImage screen. Protein amounts were quantified from phosphorImage scans on a model 860 STORM scanner and the ImageQuant software (Molecular Dynamics).

## Supplementary Material

A comparison of infectivity rates of HRSV with and without the F glycoprotein in different cell types. The ΔF virus utilizes the baculovirus GP64 protein for viral entry. HEp2, A549, and Vbac cells were infected with the ΔSH and ΔF viruses, both of which contain GFP. Infected cells, which were identified by GFP expression, were counted from 0 to 30 hours post-infection. All cell types tested were infected with the ΔSH virus. HEp2 cells were only minimally infected by the ΔF virus, while A549 cells and Vbac cells, allowed for a robust infection with the ΔF virus.
