# Supplementary material for: Requirements for Human Respiratory Syncytial Virus Glycoproteins in Assembly and Egress from Infected Cells
Source: Adv Virol. 2011 Jul 27;2011:343408. doi: 10.1155/2011/343408 (PMC3175114; doi:10.1155/2011/343408)
Supplement: Supplementary file 1 — A comparison of infectivity rates of HRSV with and without the F glycoprotein in different cell types. The ΔF virus utilizes the baculovirus GP64 protein for viral entry. HEp2, A549, and Vbac cells were infected with the ΔSH and ΔF viruses, both of which contain GFP. Infected cells, which were identified by GFP expression, were counted from 0 to 30 hours post-infection. All cell types tested were infected with the ΔSH virus. HEp2 cells were only minimally infected by the ΔF virus, while A549 cells and Vbac cells, allowed for a robust infection with the ΔF virus. [file 343408.f1.pdf]

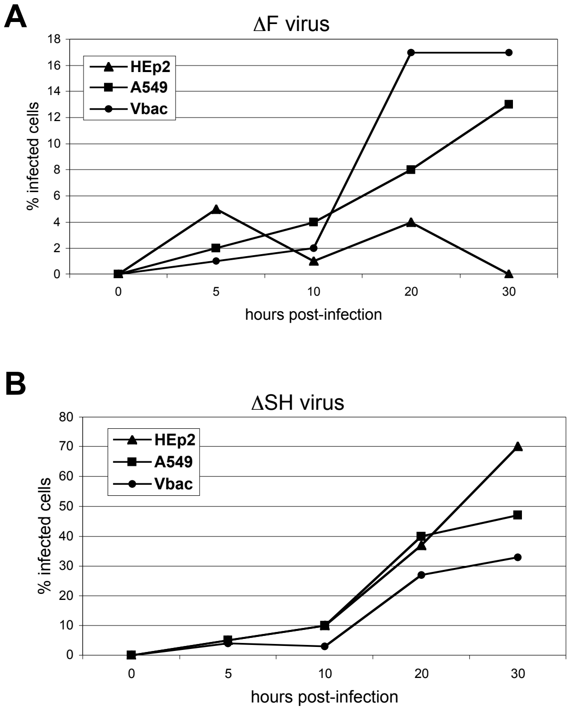

Supplemental figure 1. Comparison of infectivity rates of HRSV with and without F glycoprotein in different cell types. Cells were infected with engineered HRSV containing GFP and either with or without the F glycoprotein (ΔSH and ΔF, respectively) at a MOI 0.2. Infected cells were observed at 0, 5, 10, 20, and 30 hours post-infection and the percent of GFP expressing cells was recorded. (A) HEp2, A549, and Vbac cells infected with ΔF HRSV. (B) HEp2, A549, and Vbac cells infected with ΔSH HRSV.
